# Melatonin Mitigates Cd-Induced Growth Repression and RNA m^6^A Hypermethylation by Triggering MMR-Mediated DNA Damage Response

**DOI:** 10.3390/plants14091398

**Published:** 2025-05-06

**Authors:** Zihan Tang, Hetong Wang, Xianpeng Wang, Richard A. Ludlow, Zhouli Liu, Min Zhang, Qijiang Cao, Wan Liu, Qiang Zhao

**Affiliations:** 1 Liaoning Key Laboratory of Urban Integrated Pest Management and Ecological Security, College of Life Science and Bioengineering, Shenyang University, Shenyang 110044, China; tangzhget@163.com (Z.T.); wxp19980807@163.com (X.W.); zlliu@syu.edu.cn (Z.L.); himoli12340@163.com (M.Z.); caojiang2010@126.com (Q.C.); 2 College of Agriculture, Heilongjiang Bayi Agricultural University, Daqing 163000, China; 3 School of Biosciences, Cardiff University, Sir Martin Evans Building, Museum Avenue, Cardiff CF10 3AX, UK; ludlowra@cardiff.ac.uk; 4 Northeast Geological S&T Innovation Center, China Geological Survey, Shenyang 110034, China; 5 College of Agriculture and Biological Sciences, Dehong Normal University, Mangshi 678400, China; liuwan63@163.com

**Keywords:** Cd toxicology, RNA m^6^A methylation, DNA repair, cell cycle, Arabidopsis

## Abstract

Melatonin (MT) has been found to mitigate cadmium (Cd) toxicity with negligible environmental risks. It remains poorly understood as to how MT mitigates Cd-induced growth repression and regulates RNA m^6^A methylation. We aimed to elucidate the effect of MT on growth repression and RNA m^6^A methylation in Arabidopsis (*Arabidopsis thaliana*) exposed to Cd stress. MT mitigated, on average, 13.96% and 8.42% of growth repression resulting from Cd and mismatch repair (MMR) deficiency. The ameliorative effect on Cd stress was reduced by 70.56% and 34.23% in *msh2* and *msh6* mutants, respectively. With distinct dose–effect relationships, m^6^A hypermethylation responded to Cd stress rather than Cu stress, which was further elevated in MMR-deficient seedlings. MT reduced m^6^A levels by 22.98% even without stress induction, whereas the depressed m^6^A levels in MMR-deficient seedlings, greatly exceeding those in the WT. The “writer” and “eraser” gene expression responsible for m^6^A methylation was reduced with the concentration of stresses due to MT, but *VIR* and *ALKBH9B* no longer responded to Cd stress in *msh2* and *msh6*. Despite the remarkable repression, MMR gene expression was regularly promoted by MT under Cd and Cu stress. Our study provides novel insights into the molecular mechanisms underlying the restorative effects of MT on growth repression and m^6^A methylation regulation, which shed light on Cd phytoremediation.

## 1. Introduction

Cd is a natural element in the Earth’s crust and ubiquitously exists in soils, rocks, water, and anthropogenic materials such as batteries, pigments, metal coatings, and plastics. It is a toxic, persistent, and accumulative heavy metal (HM) that is ranked as the 7th most hazardous substance and the 4th most hazardous inorganic substance by the Agency for Toxic Substances and Disease Registry [1]. Exposure to Cd causes distinct genotoxic effects, due to DNA damage including base substitutions, base–base mismatches, insertion/deletion loops, DNA adducts, DNA breaks, DNA methylations, and DNA-strand cross-links. These affect plant and human cells alike, and exposure to Cd is commonly caused by contaminated soil and water [2,3]. In order to maintain genome integrity and prevent DNA lesion transmission to daughter cells, a DNA repair system exists to recognize DNA damage in both plants and humans. Various sensors have been found to recognize different types of DNA lesions, such as MutS in the DNA mismatch repair (MMR) system, the replication protein A (RPA) complex, and the MRE11-RAD50-NBS1 (MRN) complex [4,5]. The DNA MMR system, which is responsible for repairing base–base mismatches, insertion/deletion loops, interstrand cross-links (ICLs), and double-strand breaks (DSBs), is considered to be a biomarker for Cd stress, and it activates the DNA damage response (DDR) to trigger DNA repair pathways dependent on the DDR-regulated cell cycle stage [6,7,8,9,10,11,12,13]. Therefore, the activity of the DNA MMR system is used as a proxy of Cd stress response, through regulating Cd-induced DNA damage sensitivity [10].

N^6^-methyladenosine (m^6^A), as the best-charactered mRNA internal modification, was first discovered in 1974 [14]. METTL3 and METTL14 in mammals and MTA and MTB in plants are the “writers” for m^6^A methylation, and FTO and ALKBH5 in mammals, and ALKBH9B and ALKBH10B in plants, function as the “erasers” of m^6^A [15,16,17,18,19]. Histone H3 trimethylation is responsible for the recruitment of the writer complex by interacting with METTL14, favoring m^6^A modification on the mRNA coding sequence (CDS) and a 3′ untranslated region (3′UTR) [20]. The m^6^A-containing mRNA are enriched in important intracellular activities, and clustered m^6^A sites appear dynamic, responding to stimuli and stress [21,22]. Transcriptome-wide m^6^A hypermethylation was found in response to Cd stress in a wide range of organisms, including rats *(Rattus norvegicus)*, rice (*Oryza sativa* L.), soybean (*Glycine max* (L.) Merr.), barley (*Hordeum vulgare* L.), and more; these m^6^A-modified coding or noncoding RNAs are widely recognized as biomarkers of diseases and stresses [23,24,25,26,27,28,29,30]. However, the exact role of m^6^A modification principally depends on the “readers”, such as YTHDF2 and IGF2BPs, which regulate mRNA degradation and stability, respectively [31,32,33,34]. Rapid and transient m^6^A was linked to the effect of DNA DSBs and pyrimidine dimers (CPDs), which contributed to DNA lesion repair on the equivalent locus of m^6^A, by DNA polymerase κ and FTO [19,35].

Melatonin (N-acetyl-5-methoxytryptamine, MT) was originally discovered in the 1958 in the pineal gland. It is a naturally occurring indole that has been widely studied for its regulatory function on circadian rhythms, the immune system, and reactive oxygen species (ROS) signaling, as well as sleep, food intake, energy metabolism, and body temperature in humans [36,37,38,39,40,41]. Since being identified in 1995 in plants, MT and its homologs have received increased attention, due to the wide-ranging effects on growth, rooting, seed germination, fruit ripening, photosynthesis, osmoregulation, the immune response, and the stress response [42,43,44,45,46,47]. Furthermore, the first phytomelatonin receptor was identified to regulate stomatal closure in Arabidopsis *(Arabidopsis thaliana)*, establishing MT as a new plant hormone, rather than an interesting natural antioxidant molecule [45,48,49,50]. Many researchers have found that MT can reduce abiotic stress such as salt, drought, cold, and HMs in Arabidopsis, tomatoes (*Solanum lycopersicum* L.), cucumbers (*Cucumis sativus* L.), soybean, *Brassica napus* L., and *Phaseolus vulgaris* L., generally by means of Ca^2+^-, H_2_S-, ROS-, and/or NO-related signaling pathways [51,52,53,54,55]. Melatonin has previously been reported to mitigate Cd toxicity in plants, through promoting the ROS resistance systems, as well as HM chelators and transporters [56,57,58,59]. Furthermore, epigenetic effects of melatonin have been reported on DNA methylation and histone modifications both in mammals and plants [60,61,62,63,64,65]. Although there is increasing evidence to suggest that the regulation of m^6^A modification was a novel role for melatonin, only limited information and ambiguous descriptions of mechanisms are available to reveal how melatonin regulates Cd-induced m^6^A in plants [66,67].

In this study, wild-type (WT) and MMR-deficient Arabidopsis were used to reveal the underlying mechanisms of MT mitigating Cd-induced growth inhibition and m^6^A methylation, taking copper (Cu) stress as the contrast. Therefore, this study (1) comparatively analyzed the growth repression, m^6^A methylation, and transcriptional level of DDR-related and m^6^A-modification-related genes in WT and MMR-deficient Arabidopsis induced by Cd and Cu and (2) explored the distinct effect of melatonin on the above determinations between Cd and Cu, thereby providing novel insights into the mechanisms underlying the restorative effects of MT for Cd phytoremediation.

## 2. Methods and Materials

### 2.1. Plant Materials, Growth, and Treatment Conditions

*Arabidopsis thaliana* (Arabidopsis) seeds of WT (Col-0), *mlh1* (SALK_123174C), *msh2* (SALK_002708), and *msh6* (SALK_089638) lines were obtained from the Arabidopsis Biological Resource Center (ABRC, Columbus, OH, USA). Based on the distinct roles of MMR genes, the *mlh1* mutant exhibited dual defects in canonical mismatch repair and ATM-dependent DDR activation, whereas *msh2* and *msh6* mutants showed compromised mismatch recognition with the *msh2* mutant blocking activation of MMR-mediated DDR signaling. Approximately 1000 Arabidopsis seeds were placed in 1.5 mL tubes and surface-sterilized using 1 mL 10% (*v*/*v*) sodium hypochlorite for 3 min; then, they were sterilized with 1 mL 75% (*v*/*v*) ethanol for 30 s, and immediately rinsed with 1 mL sterile water 5 times. Then, the tube containing Arabidopsis seeds was filled with 1 mL of sterile water and placed at 4 °C for 24 h to stratify the seeds. The seeds were then sown in culture bottles containing 150 mL 1/2 Murashige and Skoog (MS) liquid medium (Basal Salt Mixture, Caisson, Colorado Springs, CO, USA) with 0.5% (*w*/*v*) sucrose (pH 5.8). For the Cd treatment, CdCl_2_ was added to the media to yield concentrations of 0, 0.5, 1, 2, and 3 mg·L^−1^, based on the minimal concentration avoiding hormesis effects and the maximum tolerable concentration for the survival of WT and MMR-deficient seedlings. Likewise, for the Cu treatment, CuCl_2_ was added to the media to yield concentrations of 0 1, 2, 3, and 4 mg·L^−1^, based on the induction of morphological variations similar to those caused by Cd stress. Across all of these heavy-metal treatments, one batch was made with 300 µM (final conc) melatonin, and another identical batch created without melatonin. The culture bottles were placed on a rotary shaker at 90 rpm in an incubator (12 h light of 3000 lx and 12 h dark at 21 ± 0.5 °C) for 7 d. Each culture bottle contained ~100 seeds and was considered as one experimental unit. All treatments and analyses were repeated in three independent replicates.

### 2.2. Morphological Measurement

The morphological characteristics of Arabidopsis seedlings were measured by scanning in a WinRHIZO Pro 2012b root scanning image analysis system (Regent Instruments, Inc., Québec City, QC, Canada). The root reduction ratio was calculated by using the following formula: (the treatment − the control)/the control × 100%. All experiments were repeated in triplicate.

### 2.3. RNA Extraction, First-Strand cDNA Synthesis, and qRT-PCR Analysis

The total RNA of the Arabidopsis seedling roots was extracted and purified using a MiniBEST Plant RNA Extraction Kit (TaKaRa, Beijing, China) from 100 mg (FW) samples. The RNA quantity and quality were measured by using a NanoDrop 2000 (Thermo Fisher Scientific, Waltham, MA, USA). The first-strand cDNA was synthesized using a TransScript All-in-One First-Strand cDNA Synthesis Super Mix Kit (TransScript, Beijing, China) from 1 μg of total RNA. Then, qRT-PCR was performed on with a CFX-96 qRT-PCR machine (Bio-Rad, Hercules, CA, USA) using a TransScript^®^ Top Green qPCR SuperMix Kit (TransScript, Beijing, China). The polyubiquitin gene *UBQ10* was used as a reference gene for signal normalization. The primers used in this study were designed using QuantPrime web tool (http://quantprime.mpimp-golm.mpg.de/; accessed on 14 April 2025) and referenced [65], and they are listed in Table S1. Relative expression levels between different treatments were calculated using the 2^−ΔΔCT^ method [68]. The qRT-PCR experiments each had three biological replicates, and three technical replicates were performed for each biological replicate.

### 2.4. RNA-m^6^A-Level Measurement

The total RNA of the Arabidopsis seedling roots was diluted to 100 ng·μL^−1^ using sterilized ddH_2_O. The RNA m^6^A level was measured using a EpiQuik™ m^6^A RNA Methylation Quantification Kit (Colorimetric; EpigenTek, Farmingdale, NY, USA) following the manufacturer’s instructions. The light absorption value of the solution at 450 nm was detected with an enzyme label (Varioskan-Flash, Thermo Scientific, Waltham, MA, USA). To quantify the absolute amount of m^6^A, a standard curve was created and the slope of the standard curve was determined using linear regression (including at least 4 data points). The amount/percentage of m^6^A in the samples’ total RNA was calculated as follows: m^6^A content (ng) = (sample OD − negative control OD)/slope. Each treatment had three biological replicates, and each biological replicate had three technical replicates.

### 2.5. Statistical Analysis

SPSS (version 23.0) was used for all statistical analyses. Results are expressed as the means ± standard deviation (SD) of three independent experiments. One-way ANOVA was used to evaluate the differences between the same cultivar among the treatments (*p* < 0.05).

## 3. Results

### 3.1. Cd- and Cu-Induced Arabidopsis Growth Repression and Its Recovery Mediated by MT

**Figure 1:**
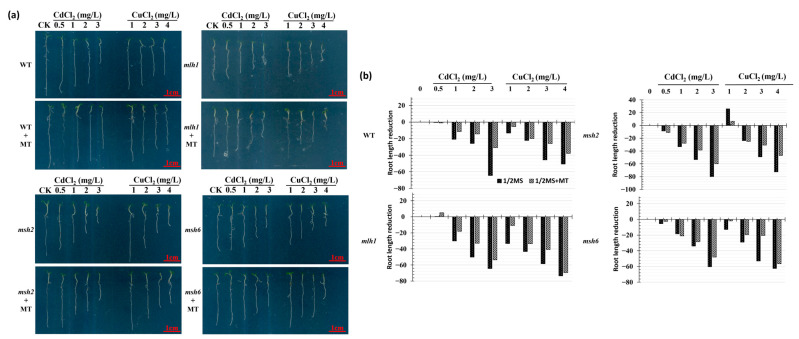
Effect of MT on growth repression induced by Cd and Cu stress between wild-type and MMR-deficient Arabidopsis. (**a**) Growth variation. (**b**) Root length reduction.

**Figure 2:**
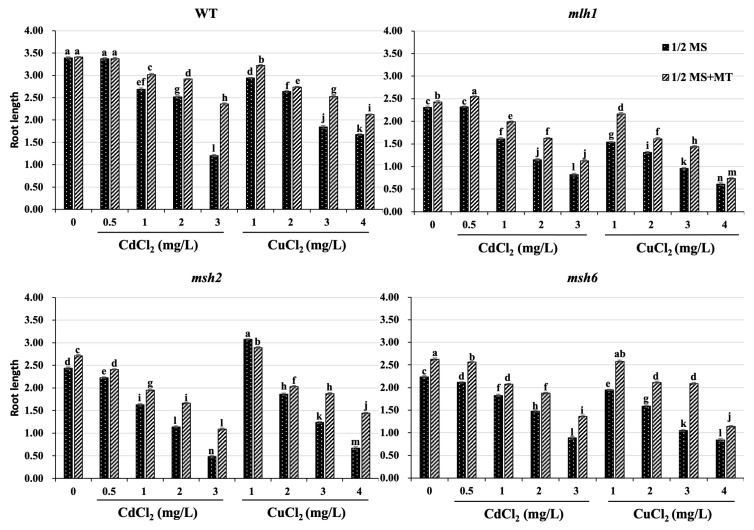
Effect of MT on root length between wild-type and MMR-deficient Arabidopsis. Different letters indicate statistically significantly differences (*p* < 0.05) among different treatments. Standard deviations were calculated with seven independent experiments, each comprising 35 seedlings.

To evaluate the effects of MT on Arabidopsis growth repression induced by Cd and Cu stress, 100 μmol·L^−1^ MT was added to WT and MMR-deficient Arabidopsis seedlings exposed to 0.5–3 mg·L^−1^ Cd^2+^ and 1–4 mg·L^−1^ Cu^2+^ stress for 5 days. There was a clear dose–effect relationship responsible for growth repression and the restorative effect of MT in both WT and MMR-deficient seedlings exposed to Cd and Cu (Figure 1). The root length of both WT and MMR-deficient Arabidopsis decreased as the concentration of Cd and Cu increased (Figure 2). The Cd-induced root reduction was greater than that of Cu at the same treatment dose, suggesting it has a stronger deleterious effect on growth than Cu. However, the MMR-deficient line exhibited an altered dose–effect relationship on root growth reduction, whereby it was broadly reduced at all concentrations. However, the root length of *mlh1* exposed to Cu had, on average, a 19.24% reduction compared to that of WT, and the roots of *msh2* exposed to Cd likewise decreased by 15.96% in length, suggesting a link between *MLH1* and *MSH2* in Cu and Cd stress responses, respectively. Furthermore, the stress resistance induced by MT varied between WT and MMR-deficient Arabidopsis. MT induced stress resistance in the WT and *mlh1* exposed to Cd, and *msh2* exposed to Cu, but the opposite results were found for *msh2* exposed to Cd and *mlh1* exposed to Cu, which further suggests that *MLH1* and *MSH2* play different roles in Cu- and Cd-stress responses. Without Cd and Cu contamination, the root lengths of WT, *mlh1*, *msh2*, and *msh6* were increased by an average of 0.65%, 5.02%, 11.11%, and 17.29%, respectively, under MT treatment (Table S2). Thus, although MT significantly reduced Cd and Cu root growth repression, its restorative effect was disparate, due to the MMR function of the seedlings. The restored root length induced by MT in MMR-deficient seedlings did not exceed that of the WT lines.

### 3.2. Restored Effect of MT on Cd- and Cu-Induced m^6^A Hypermethylation

**Figure 3:**
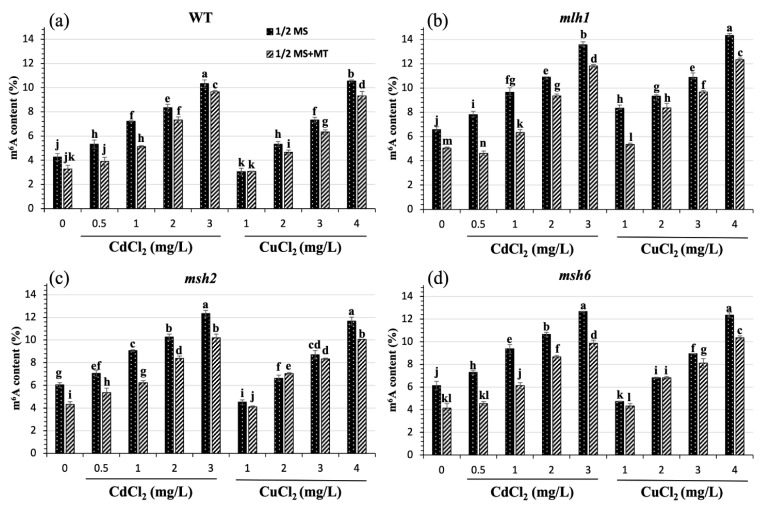
Effect of MT on m^6^A hypermethylation induced by Cd and Cu stress in wild-type and MMR-deficient Arabidopsis. The lowercase letters indicate statistically significant (*p* < 0.05) differences in gene expression for the same variety under different treatments. (**a**) The increasing Cd and Cu stress led to incremental m^6^A levels, but MT reduced Cd-induced hypermethylation significantly more than Cu-induced hypermethylation. (**b**) Although MT played an apparent role in reducing both Cd- and Cu-induced m^6^A methylation, the m^6^A levels in all the treatments of *mlh1* were promoted compared with the others. (**c**,**d**) Hypermethylation was found in *msh2* and *msh6* exposed to both Cd and Cu stress in contrast to WT. However, MT substantially repressed the hypermethylation resulting from Cd.

To further explore the different roles of MT on Cd- and Cu-induced m^6^A hypermethylation in WT and MMR-deficient Arabidopsis, transcriptome-wide RNA m^6^A levels were evaluated. Compared to the WT, a 16.32–35.15% and 9.73–63.34% increase in hypermethylation was found in MMR-deficient seedlings exposed to Cd and Cu, respectively. The highest degree of hypermethylation was found in *mlh1*, showing the DDR-dependent m^6^A hypermethylation (Figure 3). With increasing Cd and Cu stress, dose–effect relationships of raised m^6^A levels in the WT, *mlh1*, *msh2*, and *msh6* were observed. However, 22.93–28.18% hypomethylation was found in the WT, *msh2*, and *msh6* exposed to low concentrations of Cu stress, which may suggest a different mechanism between Cd- and Cu-induced m^6^A methylation. Furthermore, the incremental hypermethylation with increasing HM stress was found in of all the seedlings exposed to Cd but only *mlh1* exposed to Cu, which suggests that *MLH1* affected Cu-induced m^6^A methylation (Figure 3b).

The addition of MT to the media caused m^6^A levels to reduce in both WT and MMR-deficient seedlings exposed to Cd and Cu. The degree of reduction in Cd-induced m^6^A levels was greater than that of Cu-induced m^6^A. Indeed, MT reduced m^6^A levels by 18.6%, 25.5%, 22.8%, and 28.4% via Cd-induced hypermethylation and 9.3%, 17.8%, 5.3%, and 8.4% via Cu-induced hypermethylation, respectively in the WT, *mlh1*, *msh2*, and *msh6* (Table S3). The restorative effect of MT was significantly increased in MMR-deficient seedlings exposed to Cd, and *mlh1* exposed to Cu. However, the restorative effect of MT on *mlh1* only worked under low concentrations of Cd and Cu. Despite the significant effect of MT, the reduced m^6^A levels in MMR-deficient seedlings remained higher than that in the WT, which suggests that at the doses tested in this study, MT was not capable of fully controlling the hypermethylation caused by MMR deficiency.

### 3.3. The m^6^A-Related Gene Expression Mediated by MMR Genes and MT Responsible for m^6^A Levels

**Figure 4:**
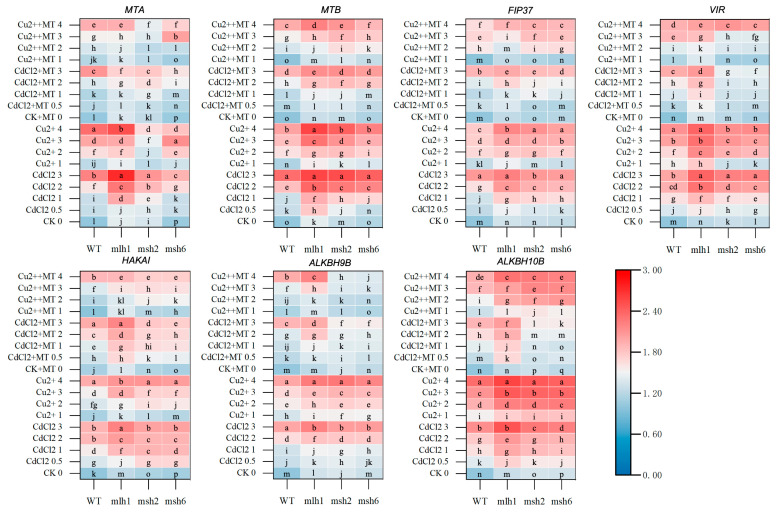
The transcriptional heat map of m^6^A-related genes between WT and MMR-deficient seedling under Cd and Cu stress. The deepening red and blue colors represent the variational transcription levels in the same cultivar. The lowercase letters indicate statistically significant (*p* < 0.05) differences in gene expression for the same variety under different treatments. MTA and MTB (the homologs of METTL3 and METTL14) are Arabidopsis mRNA methyltransferase responsible for writing m^6^A methylation. FIP37, VIR, and HAKAI, which are the accessory factors of the methyltransferase, are also considered to be m^6^A writers. ALKBH9B and ALKBH10B, the homologs of ALKBH5, are the RNA N^6^-methyladenosine demethylases, and serve as the m^6^A erasers.

To verify the above m^6^A level changes and reveal the regulatory role of MT and MMR, seven m^6^A writer and eraser genes were analyzed at the transcriptional level. The m^6^A-related gene expression in Figure 4 and Figure S1 highlights the underlying mechanism of Cd- and Cu-induced m^6^A methylation mediated by MT and MMR genes, which demonstrated that MT could reduce the overall transcriptional levels of m^6^A writer and eraser genes. Without Cd and Cu stress, very few significant changes in transcriptional levels resulted from MT application in the m^6^A writer and eraser genes, with the exception of *HAKAI*, which was elevated by MT in both WT and MMR-deficient seedlings. Also, MT promoted *VIR* expression in *mlh1* without stress induction. Additionally, all m^6^A-related gene expression was repressed by MT in MMR-deficient seedlings.

Compared with the WT, the transcriptional levels of all of the m^6^A-related gene loci were significantly elevated in *mlh1* exposed to 0–3 mg·L^−1^ Cd^2+^ and 0–4 mg·L^−1^ Cu^2+^, especially for the key “writers” *MTA* and *MTB*, which provided an explanation of significant promotion of hypermethylation in *mlh1*. Although the MT-repressed expression was no more than that in the WT, *MTA* and *MTB* expressions were repressed by MT in *mlh1* when induced by both the high and low concentrations of Cd and Cu, respectively. Likewise, with *mlh1*, the m^6^A-related gene expression was increased, particularly in *msh2* and *msh6* exposed to 0–2 mg·L^−1^ Cd^2+^. Cu stress induced varying effects on m^6^A-related gene expression, which elevated *FIP37*, *HAKAI*, *ALKBH9B*, and *ALKBH10B* in both *msh2* and *msh6*, but repressed *MTA* and *MTB* in *msh2*. However, MT restored the m^6^A-related genes back to their pre-stressed expression levels in *msh2* and *msh6*. The expressions of *FIP37*, *VIR*, *HAKAI*, *ALKBH9B,* and *ALKBH10B* in *msh2* and *msh6* were significantly reduced, especially when treated with high concentrations of Cd, which were even lower than that of the WT. However, under Cu stress, there was no clear change in expression induced by MT. Notably, the ameliorative effect of MT on the expression of *VIR* and *ALKBH9B* resulted in the dose–effect relationships being lost in *msh2* and *msh6*. In conclusion, although the expression patterns of the m^6^A-related gene loci were varied between Cd and Cu stress, and between the WT and MMR-deficient lines, the collated results above were consistent with the observed m^6^A level changes.

### 3.4. DNA-Repair-Gene Expression Mediated by MT in the Cd- and Cu-Induced DDRs

**Figure 5:**
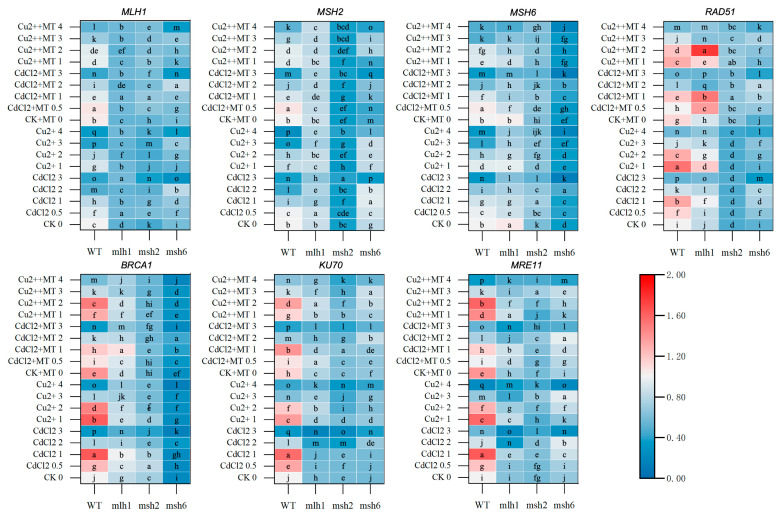
The transcriptional heat map of DNA repair genes in response to DDR between WT and MMR-deficient seedlings under Cd and Cu stress. The deepening red and blue colors represent the variational transcription levels in the same cultivar. The lowercase letters indicate statistically significant (*p* < 0.05) differences in gene expression for the same variety under different treatments. MLH1 engaged in DNA mismatch repair in the MMR system and participated in ATM-dependent DDR. MSH2, a vital protein in MMR system, formed MutS-related complex recognizing base–base mismatches, insertion/deletion loops, and interstrand crosslinks with MSH6, MSH3 and MSH7, respectively, which could recruit HR. When the DSBs occurred, RAD51 associated with BRCA1 to form Holiday junctions, which were the early indications of HR. MRE11 and KU70, which were the components of MRN and KU complexes, respectively, contributed to NHEJ responsible for DSBs repair.

To further explore the mechanism of MT action on m^6^A levels and alleviating root growth reduction, MMR genes (*MSH2*, *MSH6* and *MLH1*), homologous recombination (HR) repair genes (*BRCA1* and *RAD51*), and non-homologous end-joining (NHEJ) genes (*KU70* and *MRE11*) were examined at the transcriptional level. This also uncovered different DNA damage responses (DDRs) between Cd and Cu stress. In response to Cd and Cu treatment in WT plants, MMR gene expression reduced with increasing HM concentration; however, the dose–effect response in HR and NHEJ gene expression showed a reverse-U trend (Figure 5 and Figure S2). Furthermore, although MMR gene expression in response to Cd stress was more sensitive than that in response to Cu stress, most HR and NHEJ genes could sensitively respond to Cu stress, which indicated the distinct DDR between Cd and Cu stress. The addition of MT into WT growth mediums caused the expression of the repair-related genes, including *MLH1*, *RAD51*, *BRCA1*, *KU70*, and *MRE11*, to be upregulated without Cd and Cu stress, but the sensor-related genes remained unchanged. The repressed MMR gene expressions induced by Cd and Cu stress were recovered by MT treatment, and similar effects of MT on the DSB repair genes were only found under high-concentration-Cu stress.

In MMR-deficient lines, DNA-repair-gene expression was significantly repressed. Furthermore, both the mode and the sensitivity to respond to stresses were significantly changed. Although varying trends in expression were seen in the DNA repair genes in response to Cd and Cu stress, the gene expression levels were broadly reduced in *mlh1*. Moreover, although still reduced when compared to the WT, the effects of MT on restoring gene expression were improved for HR and NHEJ genes. However, when *MSH2* and *MSH6* were deficient, the HR gene expression was markedly suppressed compared with the WT and *mlh1*. *RAD51* expression was more strongly suppressed in *msh2*, while *BRCA1* expression was more heavily suppressed in *msh6*. Also, the effects of MT were mainly suppressed in *msh2* and *msh6* compared with *mlh1*. Furthermore, *MSH2* expression in *msh6* treated by MT was significantly reduced especially under Cd stress.

### 3.5. The DDR and Cell-Cycle-Related Gene Expression Mediated by MT Under Cd and Cu Stress

**Figure 6:**
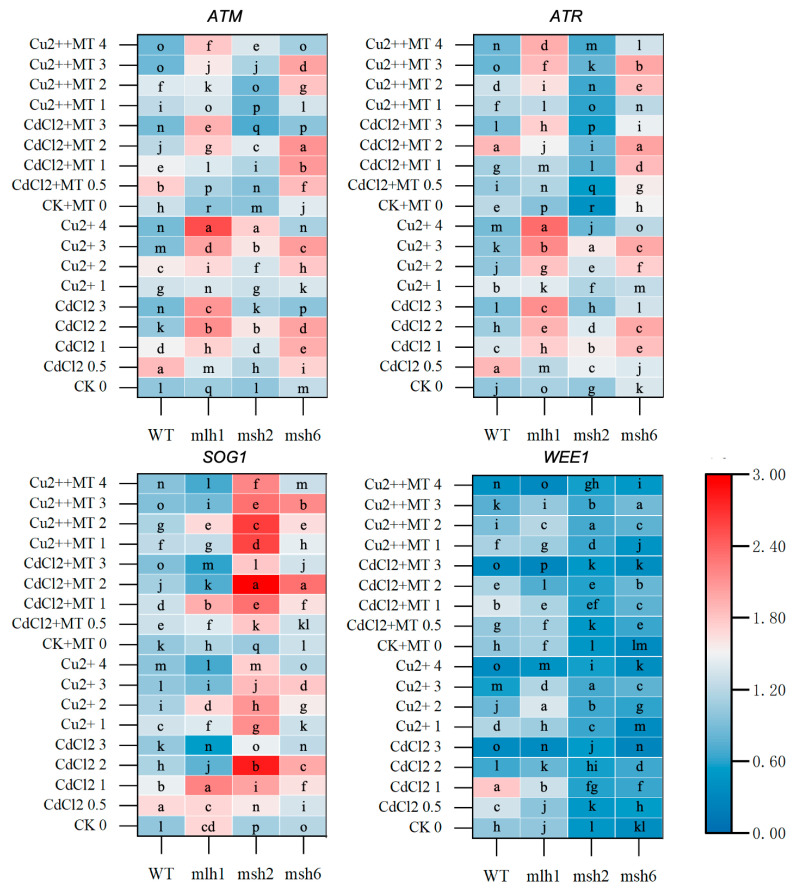
DDR gene expression in response to Cd and Cu stress between WT and MMR-deficient seedlings. The deepening red and blue colors represent the varied transcription levels in the same cultivar. The lowercase letters indicate statistically significant (*p* < 0.05) differences in gene expression for the same variety under different treatments. ATM and ATR, two protein kinases, can activate thousands of transcriptional factors, which interact with MMR, RPA complex, and MRN complex to trigger DDR. SOG1, considered to be the plant counterpart of p53, tended to be activated by ATM in the DSBs DDR. WEE1 was a kinase-regulating cell cycle, which was preferable to be activated by ATR in the DDR triggered by mismatches and single-strand breaks (SSBs).

The gene expression of four DDR key kinases was examined to identify the DDR pathway between Cd and Cu stress and the predominant role of MT on the DDR, which also contributed to revealing the mechanism of MT reducing the toxicity. The “reverse-U” dose–effect relationship was found in the expression of all DDR genes of the WT (Figure 6 and Figure S3). Moreover, although a clear effect of MT was barely found, MT could reduce the total levels of DDR gene expression. Also, the expression variations in response to Cd stress were more sensitive in the WT than those in response to Cu stress, especially on *ATR* and *WEE1* (Figure 6). When MLH1 was deficient, the expression of *ATM* and *ATR* was obviously promoted, exhibiting the gradient increasing changes. Notably, *WEE1* expression was significantly suppressed in *msh2*, but *SOG1* expression was obviously promoted, which was verified in *msh6* (Figure 6). Furthermore, prominent suppression and promotion effects of MT were found on *ATR* and *SOG1* expression, respectively.

**Figure 7:**
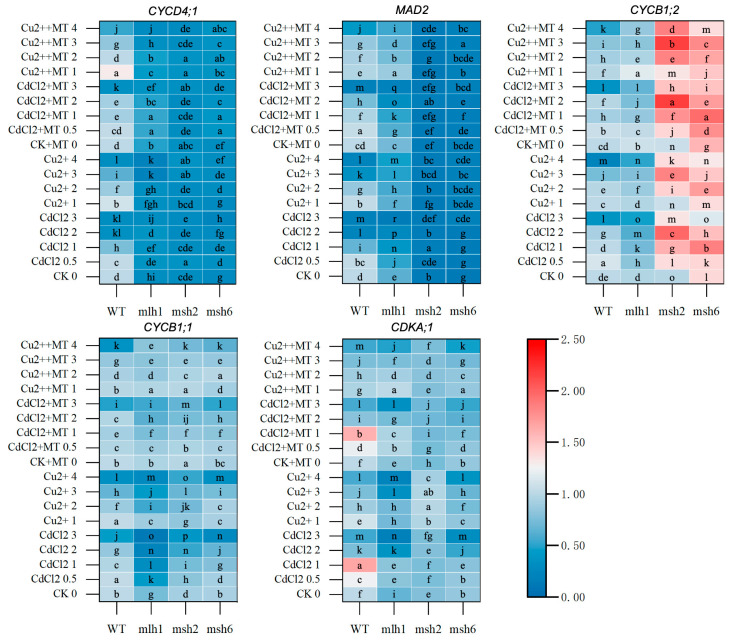
Cell-cycle-related gene expression mediated by Cd- and Cu-induced DDR between WT and MMR-deficient seedlings. The deepening red and blue colors represent the variational transcription levels in the same cultivar. The lowercase letters indicate statistically significant (*p* < 0.05) differences in gene expression for the same variety under different treatments. Although expressed throughout the cell cycle, CYCD4;1, a member of D-type cyclin, was primarily responsible for G1-S transition. CDKA;1 was a cyclin-dependent kinase responsible for both the G1-S and G2-M transitions. CYCB1;1 and CYCB1;2, which were classified as B1-type cyclins, controlled the G2-M transition in cell cycle regulation, whereas CYCB1;2 was more abundant in the late phase. MAD2, whose overexpression led to mitotic arrest, was the mitotic checkpoint protein.

Further, cell-cycle-related gene expression was analyzed for the downstream identification of the DDR, which uncovered where MT played the dominant role in the DDR pathway. The reverse-U dose–effect relationships were found in cell-cycle-related genes of the WT under Cd and Cu stress (Figure 7 and Figure S4). The expression level of *CYCD4;1* under Cu stress was obviously higher than that under Cd stress, whereas the opposite trend was present in *CDKA;1* expression. MT generally had a contradictory effect in that it suppressed gene expression under low-concentration stress conditions but promoted that under high-concentration stress conditions, with the exception of *CYCD4;1* expression under Cu stress.

Compared with the WT, the cell-cycle-related gene expression was prominently repressed in *mlh1* under Cd and Cu stress, especially for *CYCD4;1*, while that was recovered by MT to a certain extent. In *msh2* and *msh6*, *MAD2* expression was remarkably repressed, whose levels were less than 15% in *msh2* and 17% in *msh6* on average. *CYCB1;2* expression was markedly promoted, especially under Cd stress. MSH2 deficiency led to *CDKA;1* expression at an average stable level of 76% under Cd stress, which was significantly elevated to 96% by Cu stress. In addition, the obvious promotion resulting from MT was only found in *CYCB1;1* and *CYCB1;2* expression.

## 4. Discussions

In this study, the effects and the underlying mechanisms of MT alleviating Cd-induced root reduction in Arabidopsis were gradually uncovered by analyzing the Cd-induced DDR including its upstream and downstream signaling. To improve the specificity and accuracy, Cu was taken as the contrast because of its HM characteristics and it having a different DDR compared to Cd [69]. Furthermore, a m^6^A methylation analysis was used as a novel marker replacing the DNA damage and DNA methylation examination, which could reveal not only DNA damages but also the mRNA stability and translational efficiency [70].

### 4.1. MT Promotes Root Growth by Enhancing the DDR

Although without stress induction the plant cells still suffered, ROS resulted from the respiratory chain and cellular metabolisms, leading to the damage of proteins, nucleic acids, and lipids [71]. Various endogenous antioxidants were produced against them before injuries were sustained, whereas stress signaling pathways like the DDR were necessary to recover the damages. In this study, MT promoted root growth without Cd and Cu stress, and DNA repair genes, and *ATR* and *ATM* expression, were significantly elevated, suggesting the facilitated role of MT on the DDR. Additionally, root length was markedly reduced in MMR-deficient seedlings, in which the damages resulting from ROS were increased for the defects of mismatch recognition and repair. Therefore, MT could reduce the ROS injuries produced by cellular metabolisms by promoting DDR-related gene expression.

### 4.2. MT Reduced m^6^A Hypermethylation Mediated by the DDR

The m^6^A hypermethylation could result from DNA damages produced by Cd stress and oxidative stress induced by Cu [19,23,72], which was gradually elevated with the marked dose–effect relationships in this study. MT remarkably restored the above hypermethylation and reduced the total m^6^A level without stress induction. The repressed expression of m^6^A-methylation-related genes supported the above changes. Although the restorative effect of MT on Cd-induced hypermethylation was more pronounced than that on Cu-induced hypermethylation, particularly under low-concentration stress conditions, the specific mechanisms of MT alleviating hypermethylation induced by Cd and Cu stress remained ambiguous until MMR functions were impaired. Upon the knockdown of *MLH1*, despite the overall increase in hypermethylation, the restorative effect of MT on both Cd- and Cu-induced hypermethylation was significantly enhanced. *MLH1* played a pivotal role in repair mismatch, yet it had a negligible impact on the MMR-mediated DDR signaling [11]. Consequently, DNA damages were prone to accumulate in *mlh1*, which could potently trigger the DDR, ultimately resulting in the enhanced efficacy of MT. In the case of *MSH2* and *MSH6* deficiency, remarkable differences in the restored m^6^A levels between Cd and Cu stress were discerned. Despite being compared with the WT, the restorative effect on Cu-induced hypermethylation was significantly suppressed in *msh2* and *msh6*, which resulted from the different DDR signaling triggered by Cd and Cu stress. Even though they participated in various DNA repairs, MMR genes primarily recognized base damages that were strongly induced by Cd, and predominantly triggered the ATR-mediated DDR. In the event of MMR deficiency, there was a marked production of DSBs induced by Cd, which activated the ATM-mediated DDR [9,11]. In this study, the signaling pathway was verified by the significantly promoted *ATR* and *WEE1* expression in the WT and the prominently increased *SOG1* expression in *msh2* and *msh6*. However, although Cu stress induced oxidative base damages and DSBs, it showed insensitivity in responding to growth repression as well as *ATM* and *ATR* expression. This was attributed to the presence of Cu chaperones that transported Cu^2+^ to intracellular destinations [73,74]. Therefore, in *msh2* and *msh6*, the accumulated DNA damages induced by Cd were recognized by DSB sensors like the MRN complex to trigger the ATM-mediated DDR, which elucidated the sustained effect of MT on Cd-induced hypermethylation. Nonetheless, base damages and a limited number of DSBs induced by Cu were hardly detectable by the defective MMR and the normal MRN complex, which resulted in the suppressed effect of MT on hypermethylation under 1–3 mg·L^−1^ Cu^2+^ but the normal effect under 4 mg·L^−1^ Cu^2+^. In summary, MT has the potential to mitigate m^6^A hypermethylation induced by HMs, and this process is mediated by the DDR associated with these HMs. In addition, MT could significantly suppress *VIR* expression induced by Cd and Cu stress. This suppression process was regulated by MMR-mediated DDR due to the marked repression of *VIR* expression and the loss of dose–effect relationships in *msh2* and *msh6*, which suggested the mediating role of MMR-mediated DDR on MT reducing m^6^A methylation. In the future, the mechanisms of m^6^A regulation by the DDR will be further studied through both the overexpression and knockout of MMR genes, while *VIR*, which exhibits relationships with both MMR and MT, will be validated by analyzing its expression in MMR overexpression/knockout mutants under MT-induced conditions.

### 4.3. MT Restored Cd-Induced Growth Repression via the MMR-Mediated DDR

**Figure 8:**
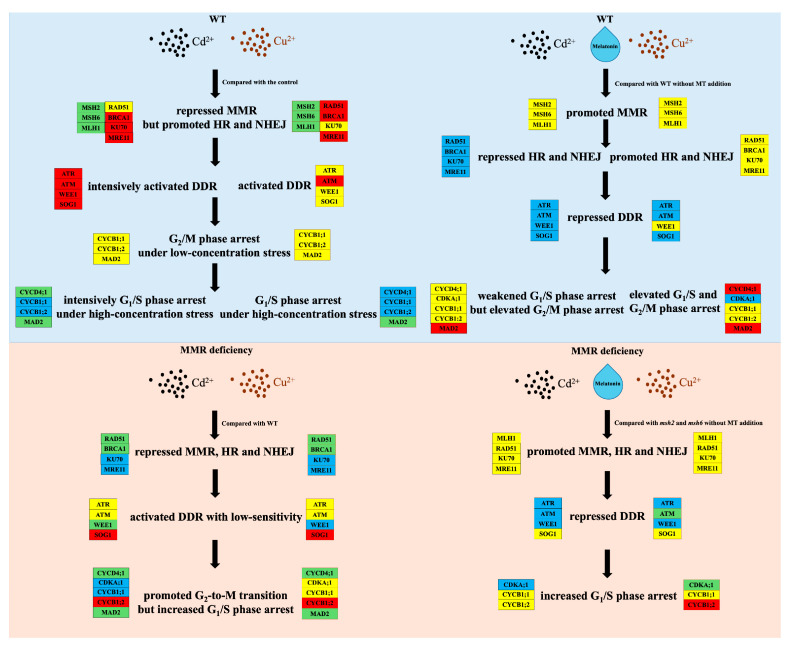
MT-mediated DDR pathways in WT and MMR-deficient seedlings under Cd and Cu stress. Both red and yellow boxes represent elevated gene expression levels, with red boxes indicating a higher level of promotion (maximal fold change >1.5). Both blue and green boxes denote reduced gene expression, with green boxes exhibiting more pronounced downregulation (maximal fold change <0.5). Black-bordered boxes highlight differential gene expression under Cd stress, while orange-bordered boxes mark Cu-induced expression changes.

Exogenous MT mitigating Cd-induced phytotoxicity and improving Cd tolerance has gradually been considered as a novel hotspot in ecotoxicology fields [56,58,75,76,77,78]. Mechanisms of MT reducing HM toxicity were concentrated on promoting a redox equilibrium and their transport [59,79], and the effect of MT promoting root growth was attributed to its involvement with photomorphogenesis signaling [49]. In this study, although MT obviously mitigated root growth reduction, the extent of restoration was notably diminished owing to deficient MMR function. The restored root length was decreased by 8.61–24.95% (average of 18.14%) and 3.57–30.71% (average of 15.33%) in MMR-deficient seedlings exposed to high-concentration Cd and Cu stress, respectively. Moreover, the shortest root lengths were observed in *msh2* exposed to Cd and *mlh1* exposed to Cu, which could be explained by the Cd- and Cu-induced DDRs. In the event of *MSH2* and *MSH6* deficiency, Cd-induced mismatches evaded the recognition of MutSα, leading to accumulative DNA lesions and the transition of DNA damage checkpoints. Mismatch repair and the subsequent HR were facilitated by G_2_/M phase arrest induced by the MMR-mediated DDR, whereas NHEJ was favored in G_1_/S phase arrest triggered by MRN complex recognizing accumulative DSBs resulting from MMR deficiency [11,13]. When compared to both G_2_/M phase arrest and endoreplication triggered by a prolonged G_2_/M phase arrest, G_1_/S phase arrest could obviously inhibit root elongation, which provided an explanation for the root growth repression in *msh2* [9,80]. Furthermore, the stimulatory effect of MT on MMR gene expression was nullified due to *MSH2* and *MSH6* deficiency, causing the weak restoration effect of MT on Cd-induced root growth repression in *msh2* and *msh6* (Figure 8). Conversely, MT could significantly alleviate Cu-induced root growth repression, especially in *msh2*, because *MSH2* and *MSH6* barely contributed to the Cu-induced DDR. To sum up, MT enhanced the MMR-mediated DDR by upregulating MMR gene expression, which relieved Cd-induced root growth repression. In the future, the mechanism will be validated against other heavy metals and inorganic pollutants across multiple plant species to determine its specificity.

## 5. Conclusions

Melatonin demonstrated significant effects in promoting plant growth and alleviating growth repression and RNA m^6^A hypermethylation induced by Cd and Cu. MT significantly upregulated the expression of DNA repair genes, *ATR* and *ATM*, which could eliminate ROS-induced DNA lesions resulting from cellular metabolism, facilitating plant growth. Also, the upregulated expression of DNA repair and checkpoint genes not only enhanced DNA repair function but also improved the Cd- and Cu-induced DDRs that reduced m^6^A hypermethylation resulting from DNA damage. However, *VIR* expression was regulated by the MMR-mediated DDR, leading to the distinct restoration effects on m^6^A methylation between Cd and Cu stress. MMR gene expression was prominently promoted by MT, which significantly mitigated Cd-induced root growth repression due to the Cd-induced DDR predominately mediated by MMR.

## Data Availability

The original contributions presented in the study are included in the article/Supplementary Material, further inquiries can be directed to the corresponding author.

## Supplementary Materials

The following supporting information can be downloaded at https://www.mdpi.com/article/10.3390/plants14091398/s1: Table S1. Primers used for qRT-PCR analysis in this study; Table S2. Root length and the restorative effect of MT under Cd and Cu stress in wild-type and MMR-deficient Arabidopsis; Table S3. m^6^A content and the restorative effect of MT under Cd and Cu stress in wild-type and MMR-deficient Arabidopsis; Figure S1. The transcriptional levels of m^6^A-related genes in WT and MMR-deficient seedling under Cd and Cu stress; Figure S2. The transcriptional levels of DNA repair genes in WT and MMR-deficient seedling under Cd and Cu stress; Figure S3. The transcriptional levels of DDR genes in WT and MMR-deficient seedling under Cd and Cu stress; Figure S4. The transcriptional levels of cell cycle-related genes in WT and MMR-deficient seedling under Cd and Cu stress.

## Abbreviations

Abbreviations	Full forms
DDR	DNA damage response
MT	Melatonin
MMR	Mismatch repair
HMs	Heavy metals
RPA	Replication protein A
MRN	MRE11-RAD50-NBS1
ICLs	Interstrand cross-links
DSBs	Double-strand breaks
m^6^A	N^6^-methyladenosine
CDS	Coding sequence
3′UTR	3′ untranslated region
CPDs	Pyrimidine dimers
ROS	Reactive oxygen species
MS	Murashige and Skoog
SD	Standard deviation
HR	Homologous recombination
NHEJ	Non-homologous end joining
