# Supplementary material for: Melatonin Mitigates Cd-Induced Growth Repression and RNA m6A Hypermethylation by Triggering MMR-Mediated DNA Damage Response
Source: Plants (Basel). 2025 May 6;14(9):1398. doi: 10.3390/plants14091398 (PMC12073604; doi:10.3390/plants14091398)
Supplement: Supplementary file 1 [file plants-14-01398-s001.zip › plants-3600205-supplementary.pdf]

Supplemental Materials

Melatonin mitigates Cd-induced growth repression and RNA m^6A hypermethylation by triggering MMR-mediated DNA damage response

[a] Liaoning Key Laboratory of Urban Integrated Pest Management and Ecological Security, College of Life Science and Bioengineering, Shenyang University, Shenyang, 110044, PR China

[b] College of Agriculture, Heilongjiang Bayi Agricultural University, Daqing, 163000, PR China

[c] Fujian Provincial Key Laboratory of Eco-Industrial Green Technology, College of Ecology and Resources Engineering, Wuyi University, Wuyishan, 354300, China

[d] School of Biosciences, Cardiff University, Sir Martin Evans Building, Museum Avenue, Cardiff, UK

[e] Northeast Geological S&T Innovation Center of China Geological Survey, Shenyang 110000, China

[f] College of Agriculture and Biological Sciences, Dehong Normal University, Mangshi, 678400, Yunnan Province, China

[*] Corresponding author at: College of Life Science and Bioengineering, Shenyang University, Shenyang, 110044, PR China; (H. Wang)

[**] Co-corresponding author at: College of Agriculture, Heilongjiang Bayi Agricultural University, Daqing, 163000, PR China; (Q. Zhao)

E-mail addresses: hetong_wang@syu.edu.cn (H. Wang); zqiang0416@byau.edu.cn (Q. Zhao); jizhanhua12@mails.ucas.ac.cn (Z. Ji); LudlowRA@cardiff.ac.uk (Richard A. Ludlow); liuwan63@163.com (W. Liu); himoli12340@163.com (M. Zhang);

caojiang2010@126.com (Q. Cao); tangzhget@163.com (Z. Tang); wxp19980807@163.com (X Wang); zlliu@syu.edu.cn (Z. Liu);

Table S1 Primers used for qRT-PCR analysis in this study

Genes	Accession numbers	Primer sequence (5')	Primer sequence (3')
UBQ10	NM_178968.5	CTCAGGCTCCGTGGTGGTATG	GTGATAGTTTTCCCAGTCAACGTC
MLH1	NM_116983.3	GTAGTAAGGTCTTCTGTAAGGCA	TGCCATTCCAACATATGTGC
MSH2	NM_113607.4	TCTGACTAGGCGAGTTCTT	CACCTCTCCAGGGAATCA
MSH6	NM_116438.2	ATTAGTTAGAAAGGGCTATCGGG	AACAACTGCACATACTTCGC
RAD51	NM_122092.3	CGAGGAAGGATCTCTTGCAG	CTAGAACTTTATCGAGCTCCCGTG
BRCA1	NM_118225.5	GTAACCATGTATTTTGCAATGCGTG	GTGACGGATTATTCTGGCTAACG
KU70	NM_101558.4	CGAGCTTCGTGAAACCAGAGATG	GTCATATTTTCCATCATCTGCGTCAC
MRE11	NM_124806.2	GTGATACACTTCGAGTACTTGTTGC	CTGACTACTTGAAACTGCACTGG
ATM	NM_001339354.1	CATGGGGTGATACAACAA	TCCTTCAGTTAGTGCATCC
ATR	NM_123447.3	GCTTTGAATTTGCTACTCT	AAGGAAACGTCCAATGGTAG
SOG1	NM_102369.3	TCTCTTGGGATCACAGGACAG	CAAGGTCTTAAGCTCGTGGTAC
CYCD4;1	NM_125940.3	GATGAGGGCATGATTGTTGACG	CCAAACTGGTGTACTTCACAAGC
MAD2	NM_113502.3	GAAGTTGCAGAGAGTAGTGTTAGTG	CTCTTCTTCATCCCACTCGTCGTTC
CYCB1;2	NM_120697.3	GACAGATTCTGGTGATGGAGAAG	CCATTCTCTGCCTTCGAGTACTTC
CYCB1;1	NM_119913.3	CTCAAAATCCCACGCTTCTTGTGG	CACGTCTACTACCTTTGGTTTCCC
CDKA;1	NM_114734.4	CCTGTCAGGACATTTACTCATGAG	GCTTTTGGCTGATCATCTCAGC
WEE1	NM_100178.3	TGGTGCTGGACATTTCAGTCGG	CAAGAGCTTGCACTTCCATCATAG
*MTA**	NM_117144.4	GGATTGCCGTCTTCAGTCTGGTTC	GCCATCATAGCGTCAGCCATGTC
*MTB**	NM_117066.4	CGAGCTGGCTGGCTTACTGTTG	CAAGATGCGGTGCGTCTGGAG
*FIP37**	NM_115277.5	TCTCAACTCAAGCCAGCGTCAATG	TGCTGCAATGTTGTCCTGGAGTTC
*VIR**	NM_111442.7	ACGAGCAGGATCTAGCAGTGGTAG	ACTTGCACAACAGCCTCTCCAAC
*HAKAI**	NM_120194.5	GTCCTCGCCGATCTTCCTGTTG	CGCAACGAACGCAGAAGTGAAC
ALKBH9B	NM_001202617.2	ATCCGTTCTCTTGACCCAGCAC	TTTCCACCACTGAACAAGAACCAG

Continued Table S1 Primers used for qRT-PCR analysis in this study

ALKBH10B	NM_116528.6	AAACGGGAAGCTTGCAGGTGAG	TCCGCCTTGACATGACCAAAGATG

* stands for the the referenced primers.

Table S2 Root length and the restorative effect of MT under Cd and Cu stress in wild type and MMR-deficient Arabidopsis

Treatment	Concentration (mg/L)	Root length (cm)				Root length reduction (%)			
		WT	*mlh1*	*msh2*	*msh6*	WT	*mlh1*	*msh2*	*msh6*
CK	0	3.39±0.03a	2.31±0.03c	2.44±0.03d	2.24±0.02c	0	0	0	0
CdCl2	0.5	3.27±0.03a	2.32±0.02c	2.23±0.01e	2.12±0.02d	-0.56	0.43	-8.53	-5.50
	1.0	2.69±0.03ef	1.61±0.03f	1.63±0.02i	1.83±0.01f	-20.74	-30.17	-33.33	-18.43
	2.0	2.52±0.03g	1.15±0.02j	1.14±0.03l	1.48±0.03h	-25.75	-50.17	-53.34	-33.96
	3.0	1.20±0.03i	0.82±0.03l	0.48±0.02n	0.89±0.03l	-64.51	-64.50	-80.20	-60.32
CuCl2	1.0	2.94±0.01d	1.54±0.01g	3.08±0.02a	1.95±0.03e	-13.24	-33.38	26.08	-12.87
	2.0	2.64±0.02f	1.31±0.02i	1.86±0.02h	1.59±0.02g	-22.21	-43.25	-23.66	-29.00
	3.0	1.84±0.03j	0.96±0.02k	1.24±0.03k	1.05±0.02k	-45.60	-58.53	-49.24	-53.08
	4.0	1.67±0.02k	0.61±0.03n	0.67±0.03m	0.84±0.01l	-50.62	-73.51	-72.61	-62.56
CK+MT	0	3.41±0.01a	2.43±0.03b	2.71±0.01c	2.63±0.03a	0.65	5.02	11.11	17.29
CdCl2+MT	0.5	3.38±0.02a	2.55±0.01a	2.41±0.02d	2.56±0.02b	-0.41	10.39	-1.15	14.48
	1.0	3.02±0.03c	1.99±0.02e	1.95±0.01g	2.08±0.02d	-11.00	-13.94	-20.05	-7.28
	2.0	3.12±0.01d	1.63±0.02f	1.66±0.02i	1.88±0.03f	-7.99	-29.65	-31.82	-16.09
	3.0	2.36±0.03h	1.13±0.02j	1.09±0.03l	1.36±0.03i	-30.44	-51.30	-55.39	-39.05
CuCl2+MT	1.0	3.22±0.02b	2.16±0.03d	2.89±0.01b	2.58±0.02ab	-4.96	-6.32	18.41	15.06
	2.0	2.74±0.02e	1.61±0.03f	2.03±0.03f	2.11±0.01d	-19.26	-30.14	-16.85	-5.63
	3.0	2.53±0.02g	1.44±0.02h	1.88±0.02h	2.09±0.03d	-25.49	-37.75	-23.12	-6.70
	4.0	2.12±0.01i	0.74±0.02m	1.44±0.02j	1.14±0.02j	-37.35	-68.05	-40.92	-49.06

Table S3 m6A content and the restorative effect of MT under Cd and Cu stress in wild type and MMR-deficient Arabidopsis

Cultivars	Stress	Stress concentrations (mg/L)	m6A content under stress (ng)		m6A content under stress and MT (ng)		Restorative effect of MT	
			Average	SD	Average	SD		Average under the same stress
WT	CK	0	4.2557	0.2884j	3.2774	0.2994jk	23.0%	
	$CdCl_2$	0.5	5.3325	0.3397h	3.8935	0.3601j	27.0%	18.6%
		1.0	7.2221	0.0385f	5.1232	0.0971h	29.1%	
		2.0	8.3361	0.2920e	7.3324	0.2524f	12.0%	
		3.0	10.3224	0.3039a	9.6552	0.1110c	6.5%	
	$CuCl_2$	1.0	3.0562	0.2726k	3.0685	0.0350k	-0.4%	9.3%
		2.0	5.3324	0.2095h	4.6555	0.1961i	12.7%	
		3.0	7.3251	0.2284f	6.3333	0.1858g	13.5%	
		4.0	10.5424	0.0887b	9.3251	0.3541d	11.5%	
mlh1	CK	0	6.5622	0.2409j	5.0332	0.0978m	23.3%	
	$CdCl_2$	0.5	7.8026	0.2835i	4.6223	0.1837n	40.8%	25.5%
		1.0	9.6351	0.3694fj	6.3321	0.2361k	34.3%	
		2.0	10.8831	0.0345e	9.3363	0.1559g	14.2%	
		3.0	13.5632	0.2281b	11.8188	0.1099d	12.9%	
	$CuCl_2$	1.0	8.3365	0.2531h	5.3526	0.1076l	35.8%	17.8%
		2.0	9.3260	0.1119g	8.3514	0.3710h	10.4%	
		3.0	10.8778	0.3506e	9.6615	0.1289f	11.2%	
		4.0	14.3354	0.1395a	12.3333	0.1363c	14.0%	
msh2	CK	0	6.0355	0.2033g	4.3126	0.0320i	28.5%	
	$CdCl_2$	0.5	7.0515	0.2801ef	5.3566	0.2465h	24.0%	22.8%

		1.0	9.0582	0.0829c	6.2231	0.3909g	31.3%	
		2.0	10.2556	0.2755b	8.3631	0.1984d	18.5%	
		3.0	12.3355	0.2827a	10.1855	0.2323b	17.4%	
	$CuCl_2$	1.0	4.5152	0.2079i	4.1137	0.3442j	8.9%	5.3%
		2.0	6.6334	0.2645f	7.0321	0.0654e	-6.0%	
		3.0	8.6986	0.3707cd	8.3215	0.0967d	4.3%	
		4.0	11.6786	0.3518a	10.0596	0.0788b	13.9%	
msh6	CK	0	6.1255	0.3659j	4.1357	0.3989kl	32.5%	
	$CdCl_2$	0.5	7.3112	0.1898h	4.5445	0.1655kl	37.8%	28.4%
		1.0	9.3667	0.3944e	6.1233	0.2604j	34.6%	
		2.0	10.6558	0.1730b	8.6522	0.1339f	18.8%	
		3.0	12.6653	0.0312a	9.8551	0.2955d	22.2%	
	$CuCl_2$	1.0	4.7211	0.0187k	4.3351	0.2106l	8.2%	8.4%
		2.0	6.8235	0.0446i	6.8263	0.1169i	0.0%	
		3.0	8.9331	0.0104f	8.1155	0.3897g	9.2%	
		4.0	12.3413	0.2167a	10.3369	0.1728c	16.2%	

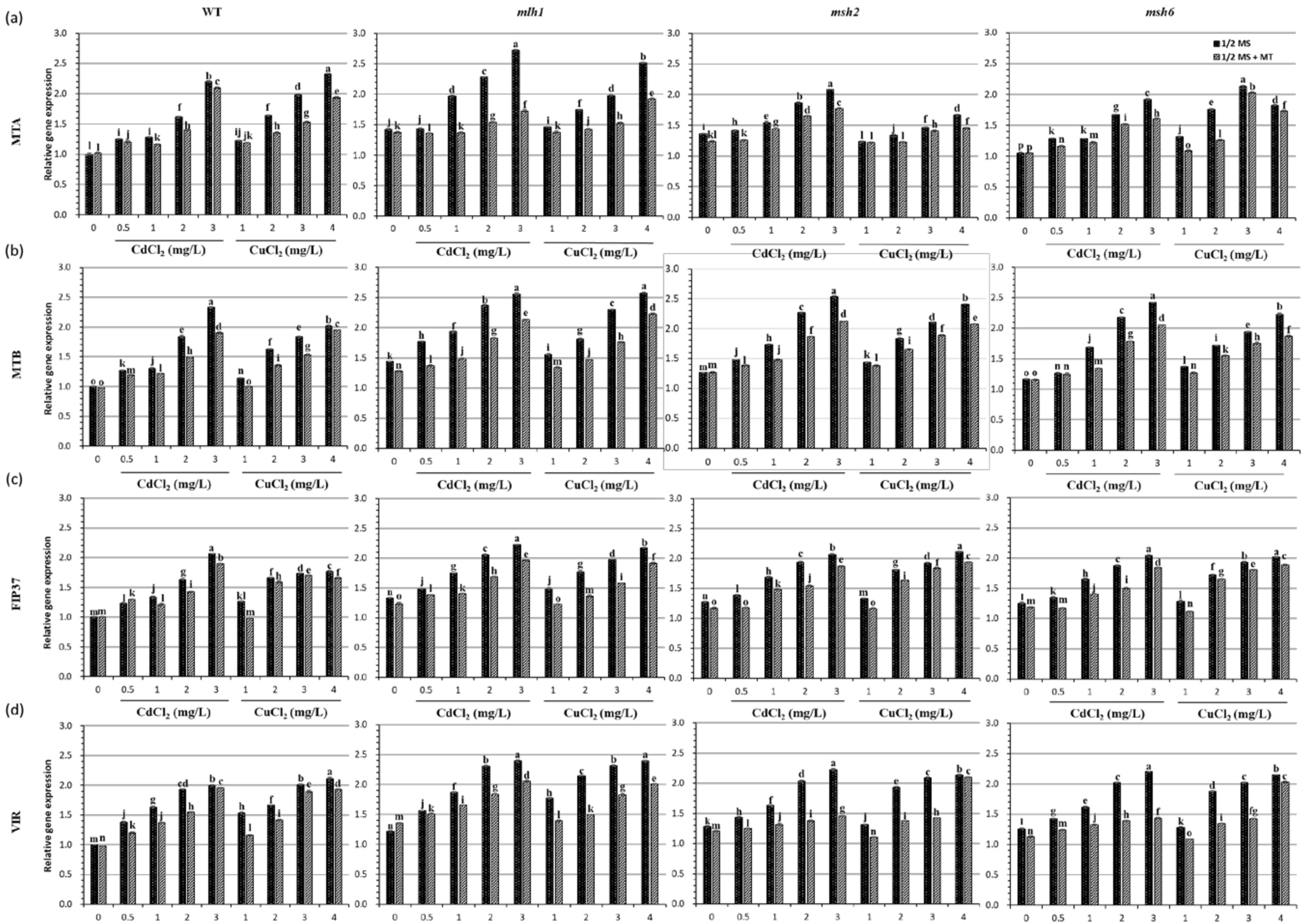
(a)
(b)
(c)
(d)
WT
mlh1
msh2
msh6
1/2 MS
1/2 MS + MT
MTA
MTB
FIP37
VIR
Relative gene expression
CdCl2 (mg/L)
CuCl2 (mg/L)

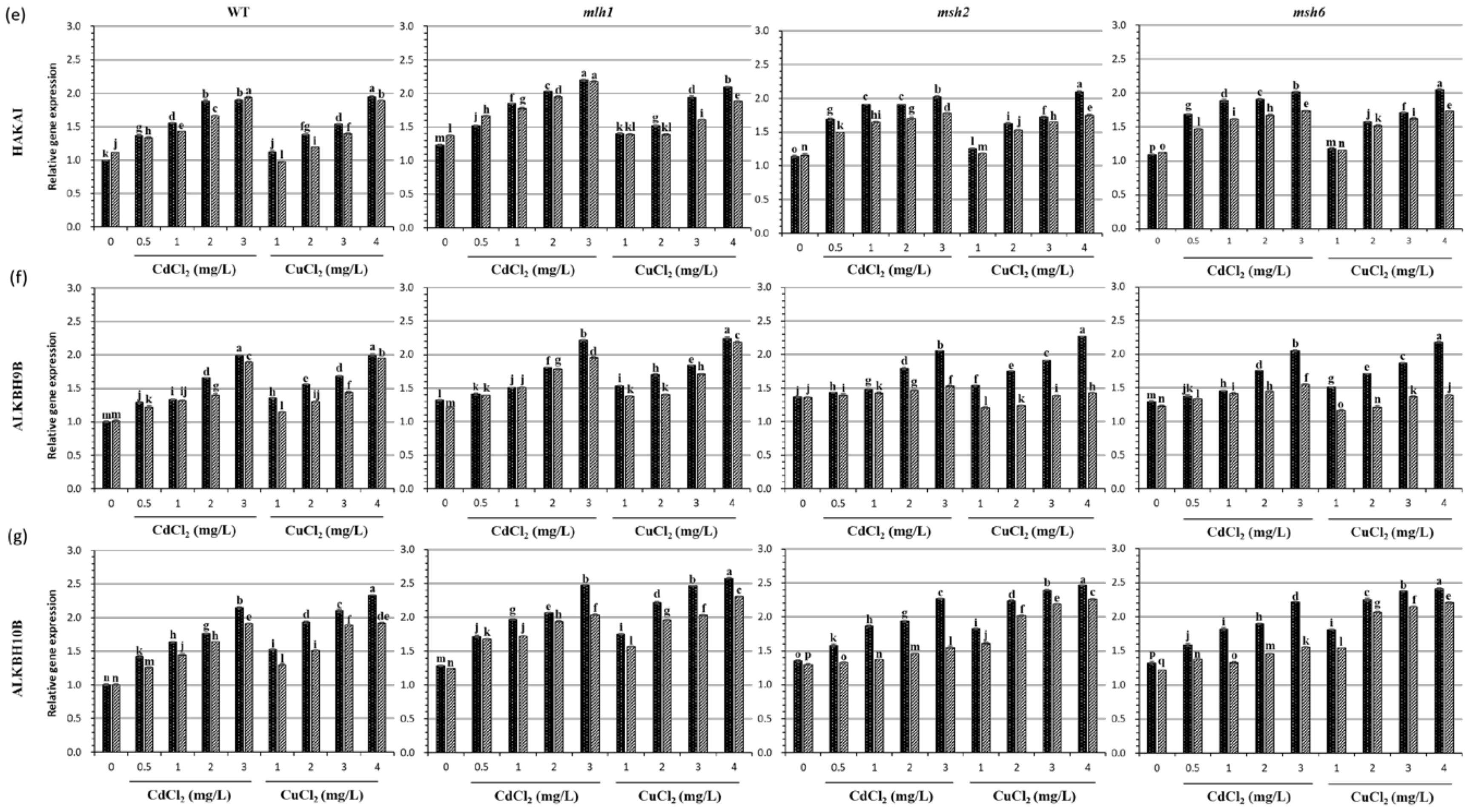

Figure S1 The transcriptional levels of m^{6}A-related genes in WT and MMR-deficient seedling under Cd and Cu stress.

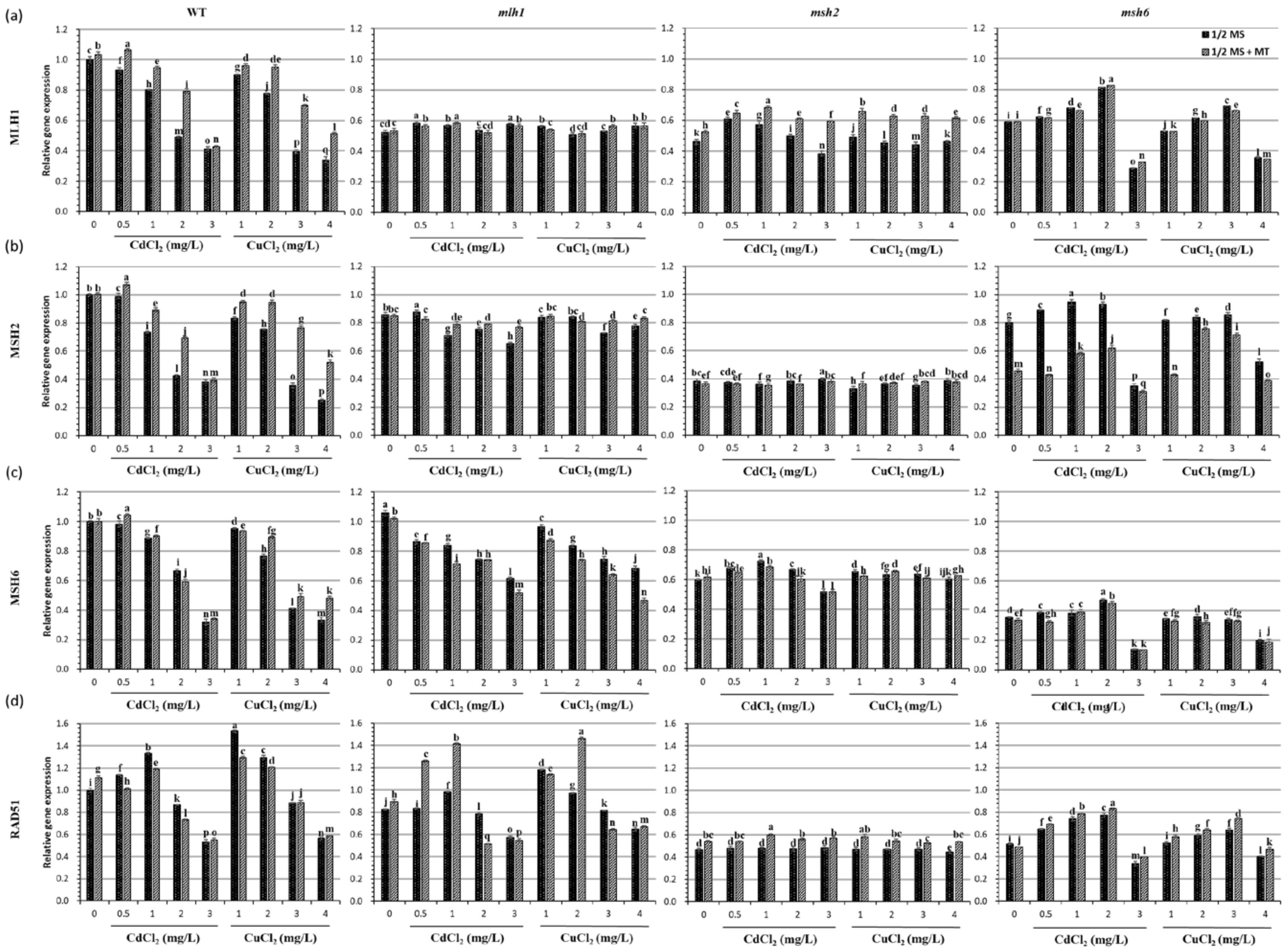
(a)
(b)
(c)
(d)
WT
mlh1
msh2
msh6
1/2 MS
1/2 MS + MT
MLH1
MSH2
MSH6
RAD51
Relative gene expression
CdCl2 (mg/L)
CuCl2 (mg/L)

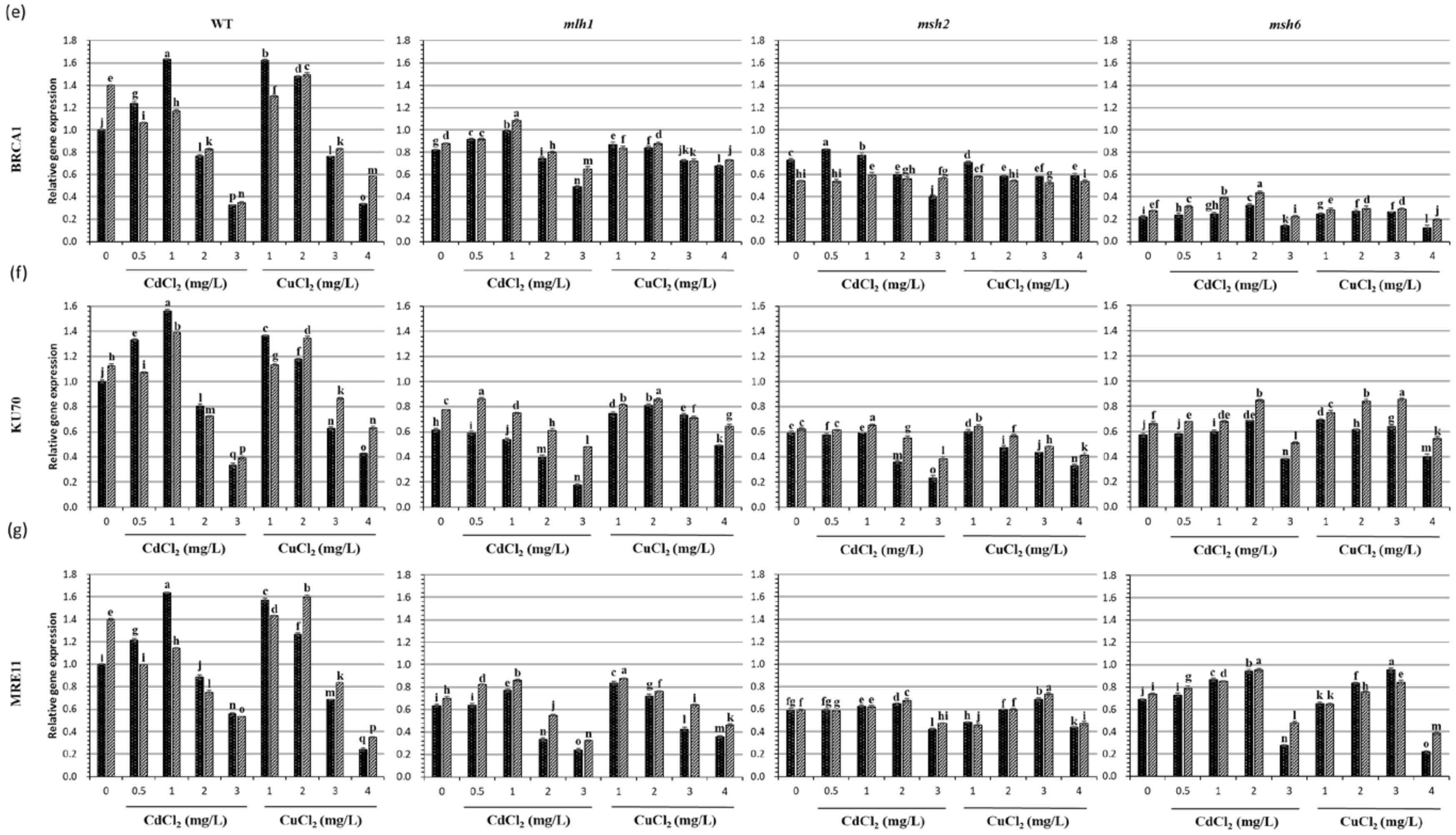

Figure S2 The transcriptional levels of DNA repair genes in WT and MMR-deficient seedling under Cd and Cu stress.

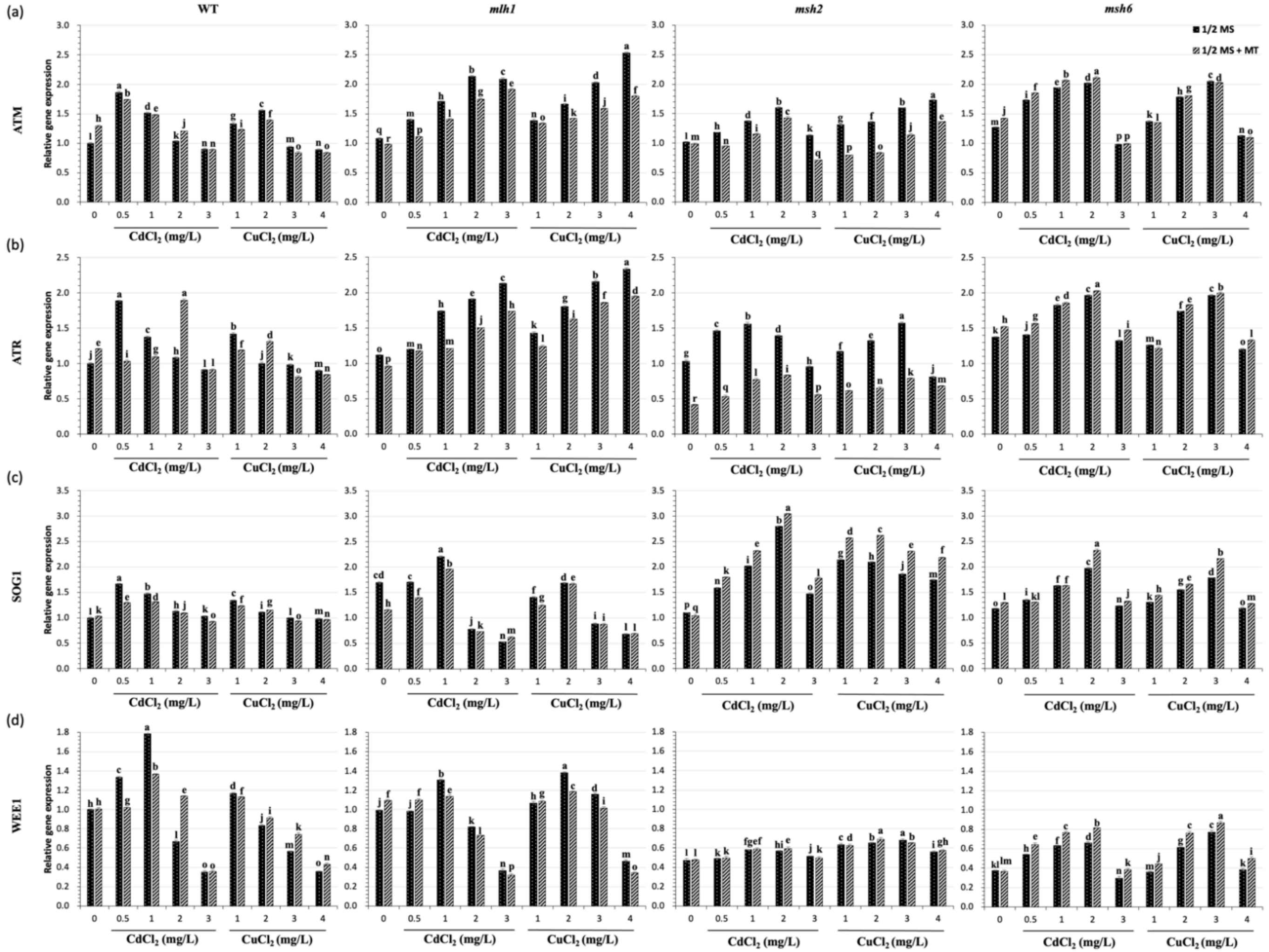

Figure S3 The transcriptional levels of DDR genes in WT and MMR-deficient seedling under Cd and Cu stress.

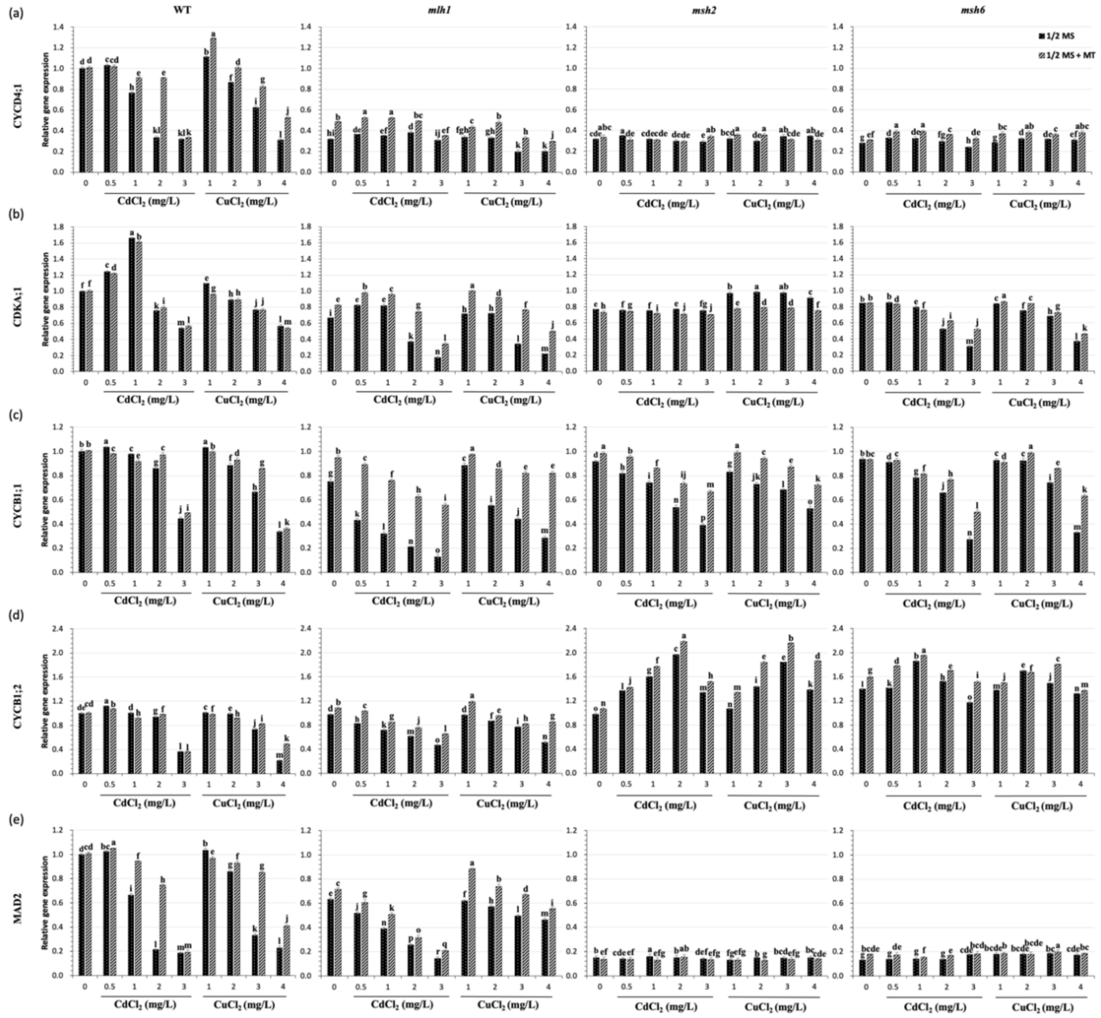

Figure S4 The transcriptional levels of cell cycle-related genes in WT and MMR-deficient seedling under Cd and Cu stress.
